# Impacts of rs7528684 (−169T/C) on *FCRL3*, *FOXP3*, and *IL-35* gene expressions and IgG-RF correlation: insights into the pathogenesis of rheumatoid arthritis

**DOI:** 10.3389/fgene.2025.1720056

**Published:** 2026-01-02

**Authors:** S. Mohamed Muzammil, S. Asha Devi

**Affiliations:** Department of Biomedical Sciences, School of Bio Sciences and Technology, Vellore Institute of Technology, Vellore, Tamil Nadu, India

**Keywords:** *FCRL3*, rs7528684, regulatory T cells, NF-κB, *FOXP3*, *IL-35*

## Abstract

**Background:**

The Fc receptor-like protein 3 (*FCRL3*) gene encodes a transmembrane receptor that is predominantly expressed on the surfaces of regulatory T cells (Tregs). The single-nucleotide polymorphism (SNP) rs7528684 (−169T/C) in the *FCRL3* gene has been hypothesised to enhance its expression, which in turn causes dysfunction of Tregs and results in loss of self-tolerance as well as diminished production of anti-inflammatory cytokines. This process triggers rapid proliferation of the autoreactive T cells and other immune cells, exacerbating the progression of rheumatoid arthritis (RA) and other autoimmune diseases.

**Methods:**

In the current study, we screened the *FCRL3* SNP rs7528684 to assess its association with the risk of RA in the Indian population. The screening was performed using high-resolution melting analysis, and the findings were confirmed via Sanger sequencing. We further analysed the impacts of rs7528684 on *FCRL3*, forkhead box protein 3 (*FOXP3),* and interleukin-35 (*IL-35*) gene expressions using quantitative real-time polymerase chain reaction. The gene expression correlation between *FCRL3* and *FOXP3* mRNA expression was analysed using Spearman’s rank correlation coefficient (ρ) analysis. Concurrently, the inflammatory serological biomarkers immunoglobulin G rheumatoid factor (IgG-RF) and C-reactive protein were evaluated in the RA patients.

**Results:**

The *FCRL3* SNP rs7528684 C/C genotype was significantly associated with increased risk of RA in the Indian ethnicity (*p =* 0.0005)*.* Furthermore, the C allele frequency was significantly elevated within the RA group (58.6%). Similarly, RA patients carrying the C/C genotype also exhibited higher *FCRL3* mRNA expression levels than the controls (*p =* 0.0072). Additionally, *FOXP3* and *IL-35* mRNA expressions were downregulated in RA patients carrying the C/C genotype. The *FCRL3* mRNA expression demonstrated a significant negative correlation with *FOXP3* mRNA expression (ρ = - 0.5526, *p* = 0.0094, R^2^ = 0.3052) in RA patients. The results also showed positive correlations between the C/C genotype and IgG-RF-positive RA cases.

**Conclusion:**

Our findings validate that the C/C genotype of the *FCRL3* SNP rs7528684 is strongly associated with RA in the Indian ethnicity. This genotype is characterised by positive IgG-RF and upregulated *FCRL3* and downregulated *FOXP3* and *IL-35* as key anti-inflammatory cytokines. Concurrently, *FCRL3* and *FOXP3* mRNA expression levels are inversely correlated.

## 1 Introduction

Rheumatoid arthritis (RA) is a multifactorial autoimmune disorder characterised by persistent inflammation leading to irreversible joint deformities, cartilage destruction, bone erosions, and disability (Said et al., 2021; Lin et al., 2020). The global prevalence of RA is approximately 0.4%–2%; its prevalence in India is known to be approximately 0.7%, with women being 2–3 times more at risk than men (Lin et al., 2020; Muravyev, 2018; Bagepally et al., 2023). Understanding the aetiology of RA is complex owing to its multiple causative factors, including environment, genetics, age, sex, and lifestyle (Liao et al., 2009; Ye et al., 2021; Goronzy et al., 2010). The genetic contribution to RA is approximately 60%, underscoring the importance of genetic variants within genes that are critically involved in the pathogenesis of RA (De Almeida et al., 2011; Van Der Helm-van Mil et al., 2010). Over the past few decades, the associations between non-HLA genes and RA have been less studied than with HLA genes. Therefore, studies on non-HLA genes are in higher demand (Lin et al., 2016). The Fc receptor-like protein 3 (*FCRL3*) is a member of the Fc receptor-like molecule (FCRL) family (*FCRL1-6*, *FCRLA*, and *FCRLB*) and is located on chromosome 1q21-23; it encodes a large family of receptor proteins that share sequence homology with other receptors and bind to the Fc regions of immunoglobulins (Bajpai et al., 2012; Matos et al., 2021; Rostamzadeh et al., 2018). These gene clusters are categorised based on their cytoplasmic motifs, differential cell-surface expressions, and binding abilities to the Fc regions of immunoglobulins (Li et al., 2014; Davis, 2007). FCRL3 is an immunoregulatory protein that is mainly expressed on the CD4^+^ FOXP3^+^ regulatory T cells (Tregs) and in specific subsets of conventional CD4^+^ T cells and B cells to a lesser extent (Zhang et al., 2020; Davis, 2007; Bajpai et al., 2012). The immunoregulatory role of the FCRL3 protein is attributable to specific motifs found in its cytoplasmic region, namely, immunoreceptor tyrosine-based activation motifs (ITAMs) and immunoreceptor tyrosine-based inhibition motifs (ITIMs) (Davis, 2007). These motifs downregulate the signal transduction pathways involved in T-cell receptor (TCR)-mediated Treg proliferation and activation. The single-nucleotide polymorphisms (SNPs) in the *FCRL3* gene lead to dysfunction and loss of self-tolerance in the Tregs. This, in turn, induces abnormal proliferation of autoreactive T cells and other immune cells, contributing to the development of RA and other autoimmune diseases such as multiple sclerosis (MS), systemic lupus erythematosus (SLE), Graves’s disease, and pancreatitis (Bajpai et al., 2012; Agarwal et al., 2020).

The SNP rs7528684 (−169T/C) present at the promoter site of the *FCRL3* gene enhances the binding affinity of the transcription factor nuclear factor kappa B (NF-κB) toward the regulatory region of the gene, which helps in rapid transcription of *FCRL3* (Report, 2007; Gibson et al., 2009; Martínez et al., 2006). Overexpression of *FCRL3* impairs the normal immunosuppressive functions of the Tregs by dysregulating their TCR signalling (Agarwal et al., 2020; Zhang et al., 2020; Bajpai et al., 2012). Tregs are crucial orchestrators of immune tolerance and exert potent suppressive functions to prevent autoimmunity. The immunosuppressive abilities of the Tregs are closely associated with the expression of the forkhead box protein 3 (*FOXP3*) gene. FOXP3 is a transcriptional factor that is uniquely expressed in Tregs and regulates the expressions of genes such as interleukin-35 (*IL-35*), transforming growth factor beta, and interleukin-10, which are involved in the immune-suppressing activities of Tregs (Zheng and Rudensky, 2007). These cytokines downregulate the autoreactive T cells, B cells, and other immune cell populations to maintain immune homeostasis and prevent autoimmunity (Attias et al., 2019). Emerging evidence suggests that the FCRL3 protein, particularly when overexpressed owing to the SNP rs7528684, could compromise the immune-suppressive capacity of Tregs. This partly occurs through the dysregulation of TCR signalling (Sakaguchi et al., 2008). Overexpressed FCRL3 protein is involved in the inactivation of protein tyrosine kinases such as LCK and ZAP70, which are pivotal enzymes in the TCR signalling pathway. Activation of these kinases is indispensable for robust TCR signalling, which is in turn required for optimal FOXP3 production and Treg function maintenance. However, TCR signalling induces epigenetic modifications that facilitate FOXP3 production (Josefowicz et al., 2012; Kochi et al., 2005; Mkaddem et al., 2017; Fernández-Aguilar et al., 2023). Disrupted TCR signalling caused by *FCRL3* overexpression leads to downregulation of *FOXP3* (Swainson et al., 2010; Bajpai et al., 2012; Moran et al., 2011; Brownlie et al., 2018; Xue et al., 2019); this downregulated *FOXP3* affects the downstream effector molecules such as IL-35 and other immunosuppressive cytokines that are directly regulated by *FOXP3*. IL-35 is a heterodimeric cytokine composed of P35 (also known as IL-2α) and Epstein–Barr virus induced gene 3 (*EBI3*) subunits that plays a significant anti-inflammatory role (Xue et al., 2019). IL-35 actively suppresses the autoreactive T cells (Schmidt et al., 2012). Therefore, dysregulated TCR signalling in the Tregs caused by the *FCRL3* SNP rs7528684 may downregulate the *FOXP3* and *IL-35* genes, which could exacerbate the severity of RA. Based on this mechanistic rationale, we aimed to explore the link between RA and the *FCRL3* SNP rs7528684 (−169T/C) in the Indian ethnicity and its subsequent impacts on the mRNA expression patterns of *FCRL3*, *FOXP3*, and *IL-35* (*EBI3* and *P35*) genes. Concurrently, we investigated its correlations with established RA serum biomarkers, namely, immunoglobulin G rheumatoid factor (IgG-RF) and C-reactive protein (CRP), and their concentrations in the serum samples of RA patients.

## 2 Materials and methods

### 2.1 Study participants and sample collection

A total of 226 RA patients and 239 healthy volunteers (aged between 30 and 60 years) recruited at Sri Narayani Hospital and Research Centre, Vellore, Tamil Nadu, India, participated in the study. The participant groups included patients who had travelled from various parts of India for RA treatment at the hospital. A *post hoc* power analysis was performed using the G^*^Power software (v3.1.9.7) to assess the study’s adequacy. Based on the cohort of 465 subjects, including both RA cases and controls, and the results of a two-tailed chi-square test (χ^2^) with α = 0.05, the calculated power was 0.99 (∼99%). This indicates that the sample is adequate for SNP screening and that there is reduced risk of type II errors. The samples were collected in according with the criteria of the American College of Rheumatology (ACR) and European League Against Rheumatism (EULAR). Patients with other comorbidities were excluded from the study. The study was approved by the Institutional Ethical Committee of the hospital (IEC/IRB No.29/08/07/2022). After obtaining informed consent from all participants, peripheral blood samples were collected by a phlebotomist using ethylenediaminetetraacetic acid (EDTA) as the anticoagulant. Approximately 5 mL of each blood sample was processed for whole genomic DNA isolation using Miller’s salting-out method, which is a widely used non-toxic and simple approach for high-quality DNA isolation from blood samples (Miller et al., 1988; Desjardins and Conklin, 2010). For the mRNA expression analysis, an additional 5 mL of peripheral blood sample was collected from each healthy volunteer and RA patient. From the obtained blood samples, we isolated peripheral blood mononuclear cells (PBMCs) using density gradient centrifugation. The total RNA was isolated from the PBMCs using the RNAiso Plus kit (TaKaRa, United States). The purity and quantity of the extracted DNA and RNA samples were measured using a NanoDrop spectrophotometer.

### 2.2 Clinical characterisation of the study participants

The RA diagnosis of the patients was confirmed according to the 2010 ACR/EULAR classification criteria. The clinical parameters of all participants were recorded at baseline using standardised case report forms, which included details regarding the age, sex, disease duration, erythrocyte sedimentation rate (ESR), IgG-RF, anti-citrullinated peptide (anti-CCP) antibody, CRP, 28-joint disease activity score (DAS28), and use of non-steroidal anti-inflammatory drugs (NSAIDs) or disease-modifying antirheumatic drugs (DMARDs). The healthy controls included in the study had no history of autoimmune diseases (Table 1).

**TABLE 1:** Baseline clinical and serological characteristics of RA patients and healthy controls.

Characteristics	RA (n = 226)	Controls (n = 239)
Sex (female/male)	144/82	132/107
Age (mean ± SD)	50 ± 11	40 ± 15
Duration of disease (years, mean ± SD)	3 ± 1	NA
ESR positivity, *n* (%)	153 (69.8%)	NA
IgG-RF positivity, *n* (%)	193 (85.4%)	NA
Anti-CCP positivity, *n* (%)	164 (72.7%)	NA
CRP positivity, *n* (%)	190 (84%)	NA
Drugs: NSAID/DMARD	0/0	NA
DAS28 score (mean ± SD)	3 ± 1	0 ± 0

### 2.3 Amplification of the *FCRL3* gene

The DNA extracted from the blood samples was diluted to 40 ng/μL to achieve uniform concentrations across all DNA samples and used as a template for *FCRL3* gene amplification. All primers used in the study were manually designed to meet specific criteria, including optimal size, GC content, annealing temperature, and self-complementarity, to prevent primer–dimer formation. For amplification of the 430-bp target region containing the *FCRL3* SNP rs7528684 locus, we used the following primers (NCBI accession number (AN) NG_023241): forward *5′-GCG​GGG​GAT​ATA​AGG​GGT​AAG-3′* and reverse *5′-CCT​TGT​CTT​CAC​ACA​GCC​T-3′*. Each polymerase chain reaction (PCR) step comprised 3 µL of the template DNA (∼50 ng/μL), 0.5 µL of each primer (forward and reverse), 0.1 µL of PrimeSTAR HS DNA polymerase, and nuclease-free water such that the reaction mixture volume was approximately 10 µL. Table 2A shows the PCR conditions used for amplification of the SNP rs7528684 target region (430 bp). Furthermore, the amplicon length was confirmed by gel electrophoresis.

**TABLE 2:** PCR and qPCR conditions for *FCRL*3 DNA amplification and genotyping.

A. PCR conditions to amplify *FCRL3* gene						
	Initial denaturation	Denaturation	Annealing	Extension	Final extension	No. of cycles
Temperature (Tm)	98 °C	98 °C	60 °C	72 °C	72 °C	40
Duration	1 min	10 s	5 s	25 s	5 min	

B. qPCR conditions for genotyping by HRMA						
	Initial denaturation	Denaturation	Annealing	Melting curve (1-s Tm intervals)		No. of cycles
Temperature (Tm)	95 °C	95 °C	58 °C	75–85 °C		45
Duration	5 min	15 s	40 s	5 s		

C. qPCR conditions to quantify gene expression						
	Initial denaturation	Denaturation	Annealing	Melting curve (1-s Tm intervals)		No. of cycles
Temperature (Tm)	95 °C	95 °C	60 °C	65–85 °C		39
Duration	30 s	10 s	30 s	5 s		

### 2.4 Genotyping of the SNP rs7528684

High-resolution melting analysis (HRMA) is a robust post-PCR genotyping technique used for SNP screening that utilises a fluorescent dye. This dye intercalates into the double-stranded DNA (dsDNA) during amplification; then, as the temperature increases gradually, the dsDNA denatures into single-stranded DNA (ssDNA). This unwinding process releases the intercalated dye, resulting in a distinct reduction of fluorescence that can be precisely monitored by the system to generate a high-resolution melting curve based on the characteristics of the DNA sequence. Unlike other traditional methods, the HRMA technique can detect subtle differences in the melting curve behaviour and does not require any labelled probes. For this method, PCR amplicons (diluted 1:20) of the 430-bp region containing the target SNP rs7528684 were used as the templates. Genotyping of rs7528684 by HRMA was performed on a Bio-Rad quantitative real-time PCR (qPCR) instrument. Each reaction included 2 µL of the DNA template, 5 µL of QIAGEN EvaGreen Master Mix, 1 µL of each internal primer (forward *5′-GAT​CTG​GGT​GAG​ATT​ACG​GG-3′* and reverse *5′-CAC​AGT​CAA​GGT​GTC​AAG​C-3*′) that targets the 119-bp region of the *FCRL3* DNA containing the SNP rs7528684 locus, and nuclease-free water to ensure a final volume of 10 µL. The qPCR conditions are detailed in Table 2B. To ensure accuracy, the samples were analysed in duplicates, and the resulting melt curves were interpreted using the Bio-Rad Precision Melt Analysis™ program. Samples with variations in the melt curve were validated by Sanger sequencing to confirm the presence of the specific SNP within the DNA locus (Shri Preethi and Asha Devi, 2022).

### 2.5 *FCRL3*, *FOXP3*, and *IL-35* mRNA expressions

The extracted mRNA samples from the PBMCs were converted to complementary DNA (cDNA) using the RT reagent (PrimeScript™ TaKaRa, United States) following manufacturer protocols. The reaction conditions used for cDNA synthesis were as follows: 15 min at 37 °C for primer annealing and cDNA synthesis, followed by 5 s at 85 °C for enzyme deactivation. The *FCRL3*, *FOXP3*, and *IL-35* (*EBI3* and *P35*) gene expression levels were analysed by qPCR using the cDNA and SYBR Green Master Mix (TaKaRa). Each reaction comprised 0.7 μL of the cDNA (∼1,000 ng/μL), 1 μL of each primer, 5 μL of the SYBR Green Master Mix, and nuclease-free water as needed to achieve a final volume of 10 μL. The qPCR conditions for the mRNA expression studies are summarised in Table 2C. Simultaneously, we used the mRNA expression level of a stable housekeeping gene such as glyceraldehyde-3-phosphate dehydrogenase (*GAPDH*) as a reference to normalise the target gene expressions. *GAPDH* is constitutively expressed in metabolically active cells, including PBMCs, under various physiological conditions that ensure consistent expression; hence, we utilised it as the control gene (Kozera and Rapacz, 2013; Fundel et al., 2008). The following primers were used for mRNA analyses: *FCRL3* (NCBI cDNA AN: BC028933), forward: *5′-CCC​CAA​AAG​CTG​TAC​TTC​TC-3′* and reverse: *5′-GCT​AGG​GAA​TGT​GAT​ATG​CTG-3*′; *FOXP3* (AN: NP_054728), forward: *5′-GAG​AAG​GAG​AAG​CTG​AGT​G-3′* and reverse: *5′-GGA​GCC​CTT​GTC​GGA​TGA​T- 3′*; *EBI3* (AN: NP_005746), forward: *5′-GCA​GCT​TCG​TGC​CTT​TCA​TA-3′* and reverse: *5′-CTA​CTT​GCC​CAG​GCT​CAT​TGT-3′*; *P35* (AN: NP_000873), forward: *5′-CTG​GAC​CAC​CTC​AGT​TTG​GC-3′* and reverse: *5′-GGT​GAA​GGC​ATG​GGA​ACA​TTC-3′*; *GAPDH* (AN: NP_001276674), forward: *5′-ATC​GTG​GAA​GGA​CTC​ATG​AC-3′* and reverse: *5′-GCA​GGG​ATG​ATG​TTC​TGG​A-3′.*

### 2.6 Gene expression correlation analysis

To determine the relationship between the mRNA expression levels of *FCRL3* and *FOXP3* in RA patients, we calculated the Spearman’s rank correlation coefficient (ρ) using GraphPad Prism v9.4.1. The resulting correlation coefficient and two-tailed *p-*value were used to assess the strength and significance of the monotonic relationship between these two genes. A *p-*value of <0.05 was considered to be significant. Additionally, for visualisation, a simple linear regression trend line was fitted to the scatter plot of correlation values. The coefficient of determination (R^2^) was reported to indicate the proportion of linear variance.

### 2.7 Measurements of the IgG-RF and CRP levels in RA serum samples

Serum samples were collected from the 226 RA patients by centrifuging their blood samples for 10 min at 3,000 rpm. The IgG-RF levels were measured in the serum samples using a commercially available human rheumatoid factor antibody ELISA kit (Cusabio, China). The assay was performed as per the manufacturer’s protocol by considering IgG-RF values <20 IU/L as negative and ≥20 IU/L as positive. The serum CRP levels were similarly quantified using a high-sensitivity CRP assay kit (Cusabio, China); here, CRP levels ≤1.0 mg/L were deemed to be in the normal range. The IgG-RF and CRP values were interpreted according to the ACR/EULAR criteria (Bykerk and Massarotti, 2012).

### 2.8 Statistical analysis

The allele and genotype frequencies of the *FCRL3* SNP rs7528684 were calculated for both controls and RA patients of Indian ethnicity. The associations of the variants to RA risk were assessed through the odds ratio (OR), 95% confidence interval (CI), and relative risk (RR) for all three genotypes (T/T, C/T, and C/C) using MedCalc software (version 23.0.2), and *p*-values <0.05 were considered to be significant. Statistical tools such as the unpaired Student’s t-test and one-way analysis of variance (ANOVA) were used to assess statistical differences in the mRNA expression levels across the various comparison groups for all target genes. The data were plotted using GraphPad Prism (version 10) and are presented as the mean ± standard error of the mean (SEM). The IgG-RF and CRP levels were first categorised into clinically relevant positive (+ve) and negative (-ve) groups. To minimise statistical errors, the dataset was analysed using multiple parameters, including the χ2 test for association analysis and Benjamini–Hochberg false discovery rate (FDR) correction for multiple tests. In all cases, results with *p-*values <0.05 were considered to be statistically significant.

## 3 Results

### 3.1 SNP genotyping

**FIGURE 1:**
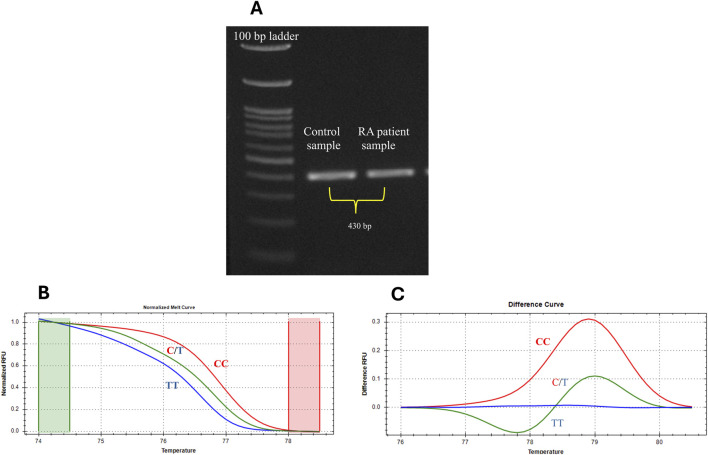
**(A)** PCR amplicons of the *FCRL3* SNP rs7528684 target region (430 bp) from controls and RA patients. **(B)** Normalised melting curves of the SNP rs7528684. **(C)** Difference curve showing three different genotypes.

**FIGURE 2:**
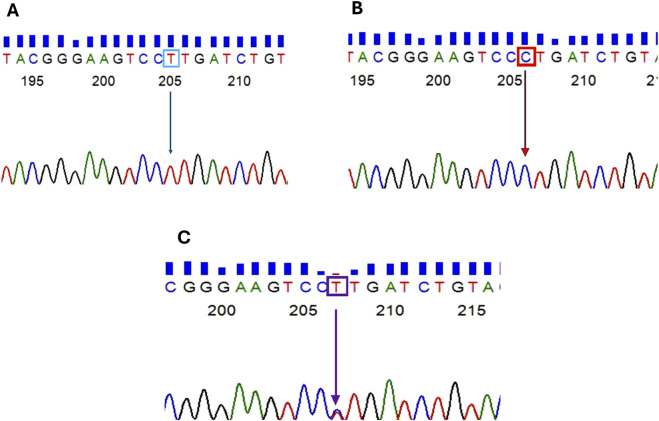
Sanger sequencing chromatogram representing the SNP rs7528684 region: **(A)** homozygous dominant T allele; **(B)** homozygous recessive C allele; **(C)** heterozygous C/T alleles.

The SNP rs7528684 (−169T/C) located within the regulatory region of the *FCRL3* gene was screened in 239 healthy controls and 226 RA patients. As an initial step, the SNP-containing region was amplified from the isolated DNA in both RA and control samples. Figure 1A illustrates the PCR product of the *FCRL3* SNP rs7528684 target region (430 bp). Furthermore, the amplicons were subjected to HRMA for genotyping. The HRMA results revealed three distinct clusters in the normalised melting curve (Figure 1B) and difference melting curve (Figure 1C), indicating the presence of the T/T, C/T, and C/C genotypes at the SNP rs7528684 locus. The samples with deviated curves were subjected to Sanger sequencing to confirm the presence of the different genotypes. The Sanger sequencing results revealed the presence of wild (T/T) and variant (C/C and C/T) genotypes in the rs7528684 locus of the *FCRL3* gene (Figures 2A–C). Table 3 indicates the genotype frequencies of the SNP rs7528684 in the control and RA patient samples. The C/C genotype was significantly more prevalent among RA patients than controls. The genotype frequencies in the controls were T/T = 41%, C/T = 31.8%, and C/C = 27.2%, while those in the RA patients were T/T = 33.3%, C/T = 18.1%, and C/C = 49.5%. A significant correlation was found between RA and the C/C genotype in the Indian population (OR = 2.63, CI = 1.78–3.86, χ^2^ = 24.583, adj *p =* 0.0003) compared to the C/T (OR = 0.47, CI = 0.30–0.73, χ^2^ = 11.541, adj *p =* 0.0012) and T/T (OR = 0.68, CI = 0.46–1.00, χ^2^ = 3.776, adj *p =* 0.0522) genotypes (Table 4). Similarly, the RR was higher for the variant C/C genotype (RR = 1.82, CI = 1.42–2.32). The genotype frequencies in the control group were tested for consistency with the Hardy–Weinberg equilibrium (HWE) using a χ2 test, where *p* ≥ 0.05 indicated no significant deviation from the HWE. Thus, a significant association was found between the *FCRL3* SNP rs7528684 (−169C) and RA in the Indian ethnicity. The C allele frequencies were 58.6% in RA patients and 43.1% in controls; the T allele exhibited corresponding distributions of 41.4% in RA patients and 56.9% in controls (Table 3).

**TABLE 3:** *FCRL3* SNP rs7528684 genotype and allele frequencies.

SNP rs7528684	f(TT)	f(CT)	f(CC)	f(T)	f(C)
Control sample (n = 239)	41.0	31.8	27.2	56.9	43.1
RA sample (n = 226)	33.3	18.1	49.5	41.4	58.6

**TABLE 4:** Association analysis between *FCRL3* SNP rs7528684 genotypes and RA risk.

Genotype	χ^2^	Odds ratio	95% confidence interval	Z-statistic	Relative risk	95% confidence interval	*p*-value	Adjusted *p*-value	False discovery rate
TT	3.7761	0.68	0.46 to 1.00	1.94	0.78	0.61 to 1.00	0.0522	0.0522	0.0522
CT	11.451	0.47	0.30 to 0.73	3.35	0.57	0.40 to 0.79	0.0008	0.0012	0.0012
CC	24.583	2.63	1.78 to 3.86	4.90	1.82	1.42 to 2.32	0.0001	0.0003	0.0004

### 3.2 *FCRL3* mRNA expression levels

**FIGURE 3:**
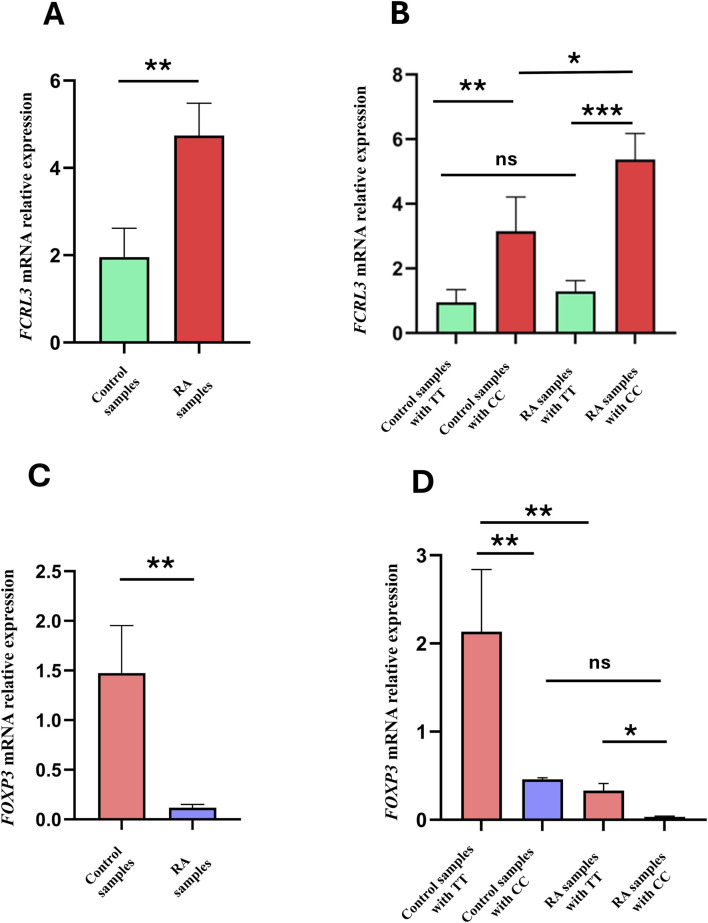
**(A)** FCRL3 relative mRNA expression levels between controls and RA patients. **(B)** Comparison of FCRL3 mRNA expression levels among different genotypes of the *FCRL3* SNP rs7528684. **(C)** FOXP3 relative mRNA expression levels in the controls and RA patients. **(D)** Comparison of FOXP3 mRNA expression levels among different genotypes of the *FCRL3* SNP rs7528684 (*p* > 0.05 = non-significant (ns), *p < 0.05, **p < 0.01, ***p < 0.001 = significant).

To study the link between the *FCRL3* SNP rs7528684 and RA pathogenesis, the mRNA expression levels of *FCRL3* were quantified in both controls and RA patients, and comparisons were carried out among the different genotypes of rs7528684. To quantify the relative expression levels, mRNA fold changes were calculated from the qPCR data using the 2^−ΔΔCt^ method, where *p-*values <0.05 were considered significant. As depicted in Figure 3A, the *FCRL3* mRNA expression levels were significantly higher (4.7 ± 0.74) in RA samples than the controls (1.9 ± 0.6, *t =* 2.803, **p = 0.0072). Figure 3B indicates the *FCRL3* mRNA expression levels within different genotypes of rs7528684. The *FCRL3* expression levels were higher in the C/C genotype (5.3 ± 0.8) than T/T genotype (1.2 ± 0.1, ***p = 0.0002) in the RA samples and in the C/C genotype of controls (3.1 ± 1.06, *p = 0.0324). These results clearly indicate higher *FCRL3* gene expressions in RA patients, particularly in those carrying the C/C genotype at the *FCRL3* SNP rs7528684 locus.

### 3.3 *FOXP3* mRNA expression levels

The impacts of *FCRL3* overexpression associated with the SNP rs7528684 (−169C) on Treg functions were assessed by measuring the *FOXP3* gene expressions in both RA patients and controls. *FOXP3* is a transcription factor that is uniquely expressed in Tregs and regulates the immune-suppressive cytokines produced by Tregs during normal immune homeostasis. Therefore, to correlate the negative influence of high FCRL3 protein levels with impaired Treg suppressive function, we measured the *FOXP3* mRNA expressions in both RA and control samples. From the qPCR results, we observed that the *FOXP3* mRNA expression levels were significantly reduced in RA patients (0.1 ± 0.03) than the controls (1.4 ± 0.4, *t =* 3.100, **p = 0.0056) (Figure 3C). Similarly, when the *FOXP3* mRNA expression levels were compared across the genotypes of rs7528684, the C/C genotype in RA patients showed significantly lower expression (0.03 ± 0.005) than the wild (T/T) genotype (2.1 ± 0.7; *p = 0.0158). In contrast, no significant difference was found in the C/C genotype between the controls and RA patients (0.4 ± 0.01; non-significant *p* = 0.8311) (Figure 3D).

### 3.4 *IL-35* (*EBI3* and *P35*) mRNA expression levels

**FIGURE 4:**
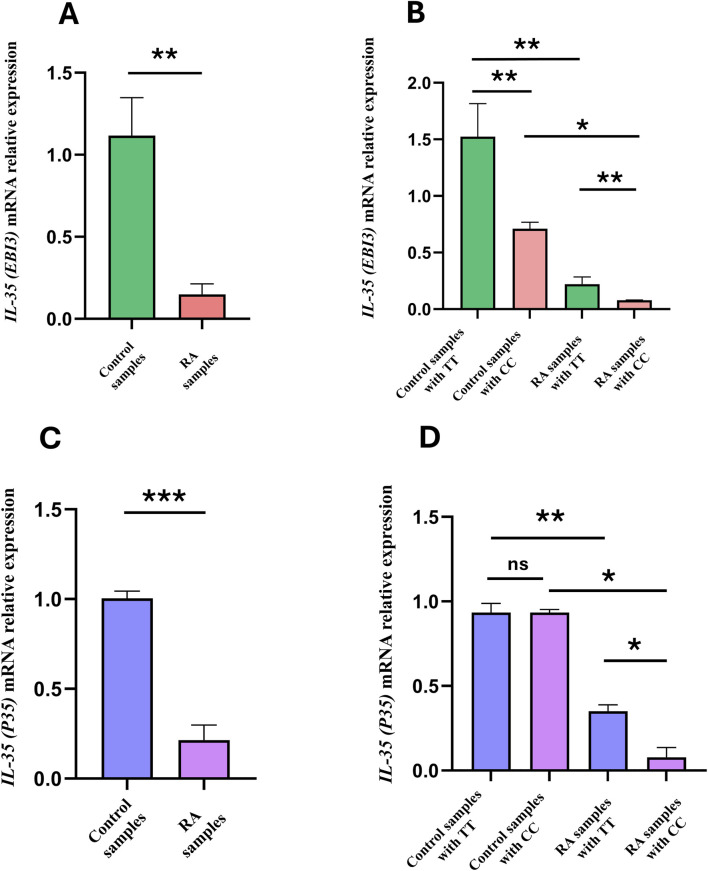
**(A)** EBI3 relative mRNA expression levels in the controls and RA patients. **(B)** Comparison of EBI3 mRNA expression levels among different genotypes of the *FCRL3* SNP rs7528684. **(C)** P35 relative mRNA expression levels between the controls and RA patients. **(D)** Comparison of P35 mRNA expression levels among different genotypes of the *FCRL3* SNP rs7528684 (*p* > 0.05 = non-significant (ns), *p < 0.05, **p < 0.01, ***p < 0.001 = significant).

Insufficient levels of immunosuppressive cytokines are recognised as a characteristic of RA. Recent studies have consistently reported that abnormal expressions of these cytokines are linked with most known autoimmune disorders, including RA, which can lead to persistent inflammation and tissue damage. IL-35 is a key immunosuppressive cytokine composed of the EBI3 and P35 subunits that reduces the autoreactive immune cells and prevents autoimmunity. To analyse the impacts of rs7528684 on suppressive cytokines, we evaluated the mRNA expression levels of *EBI3* and *P35*. As shown in Figure 4A, the RA patients exhibited a significant decrease in *EBI3* mRNA expression (0.1 ± 0.06) than the controls (1.1 ± 0.2, *t =* 4.042 **p = 0.0012). Subsequently, the mRNA levels of *EBI3* were compared among the different rs7528684 genotypes. RA patients carrying the C/C genotype exhibited significantly decreased *EBI3* mRNA expression levels (0.07 ± 0.001) than the T/T (1.5 ± 0.2, **p = 0.0047) and C/C (0.7 ± 0.05, *p = 0.0337) genotypes of the controls (Figure 4B). Similarly, the *P35* mRNA expression levels were decreased in the RA patients (0.2 ± 0.08) than the controls (1.0 ± 0.03, *t =* 8.545 ***p = 0.0001) (Figure 4C). Among the three rs7528684 genotypes, RA patients with the C/C genotype showed significantly lower *P35* expression levels (0.07 ± 0.05) than T/T (0.9 ± 0.05, *p = 0.0192) and C/C (0.9 ± 0.01, *p = 0.045) genotypes among the control samples. However, no significant difference was observed in the *P35* mRNA expression levels between the C/C and T/T genotypes in the control group (Figure 4D).

### 3.5 Inverse correlation between *FCRL3* and *FOXP3* mRNA expressions in RA

**FIGURE 5:**
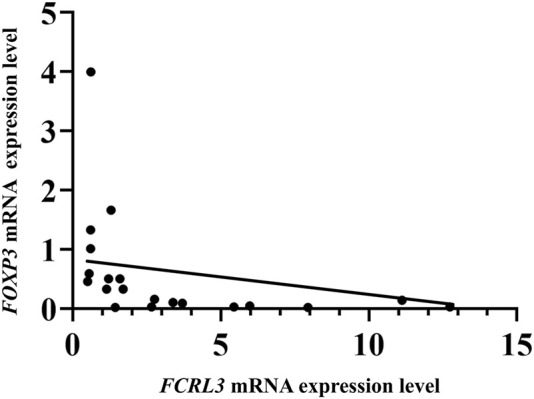
Inverse correlation between FCRL3 and FOXP3 mRNA expression levels based on Spearman coefficient (ρ = -0.5526, *p =* 0.0094) with a simple linear regression trend (R^2^ = 0.3052).

*FCRL3* and *FOXP3* mRNA expressions displayed a significant negative monotonic relationship in RA patients. The Spearman’s correlation analysis revealed a moderate-to-strong inverse correlation (ρ = -0.5526, 95% CI = -0.7997 to -0.1454, *p* = 0.0094) and linear regression trend line (R^2^ = 0.3052), confirming the inverse trend despite the modestly linear fit. The samples with elevated *FCRL3* mRNA significantly downregulated *FOXP3* expression (Figure 5). This inverse relationship aligns with the hypothesis that high FCRL3 protein levels inhibit Treg immunosuppressive functions in RA by downregulating *FOXP3* gene expression. The reduction in the FOXP3 transcription factor directly impairs IL-35 (EBI3 and P35), which is a key Treg-derived anti-inflammatory cytokine, thereby exacerbating RA progression. These data suggest that *FCRL3-*mediated suppression of *FOXP3* represents a pathogenic mechanism of Treg homeostasis disruption in RA.

### 3.6 IgG-RF and CRP levels in RA serum samples

IgG-RF and CRP are well-documented serum biomarkers that have been used to diagnose RA and are highly associated with abnormal activation of the autoreactive immune cells (Tanaka, 2025; Jiang et al., 2021). We studied the impacts of different genotypes of the *FCRL3* SNP rs7528684 on the IgG-RF and CRP levels in RA serum samples. The results revealed that the C/C genotype was significantly associated with a higher number of IgG-RF-positive cases (OR = 2.21, 95% CI = 1.09–4.82, *p =* 0.0445) than the C/T and T/T genotypes (Table 5). However, no significant links were observed between the CRP level and different SNP rs7528684 genotypes.

**TABLE 5:** *FCRL3* SNP rs7528684 genotype frequencies in RA patients stratified by RF and CRP statuses.

Inflammatory marker	Genotype of SNP rs7528684			Genotype TT vs. CT + CC		Genotype CT vs. TT + CC		Genotype CC vs. CT + TT	
	TT	CT	CC	Odds ratio (95% confidence interval)	*p*-value	Odds ratio (95% confidence interval)	*p*-value	Odds ratio (95% confidence interval)	*p*-value
RF positive (n = 193)	58 (30.1%)	34 (17.6%)	101 (52.3%)	0.51 (0.24–1.08)	1.0804	0.78 (0.31–1.96)	0.6115	2.21 (1.01–4.82)	0.0445
RF negative (n = 33)	15 (45.5%)	7 (21.2%)	11 (33.3%)						
CRP positive (n = 190)	61 (32.1%)	32 (16.8%)	97 (51.1%)	0.94 (0.44–2.01)	0.8851	0.60 (0.26–1.41)	0.2476	1.46 (0.71–3.00)	0.3035
CRP negative (n = 36)	12 (33.3%)	9 (25%)	15 (41.7%)						

## 4 Discussion

RA is a chronic autoimmune condition characterised by pain in the joints, swelling, inflammation, degradation of the cartilage, and destruction of the synovial joints, which results in permanent disability. Most autoimmune diseases are associated with proteins encoded by the immunoregulatory genes that modulate T-cell activities. *FCRL3* is one such immunoregulatory gene that encodes a transmembrane receptor expressed on the surfaces of Tregs to inhibit their proliferation. The FCRL3 receptor has two crucial cytoplasmic domains, namely, ITAMs and ITIMs, which play essential roles in regulating the Tregs (Agarwal et al., 2020). The ITIM domain recruits SHP-1 and SHP-2 as the protein tyrosine phosphatases (negative regulators of downstream signalling) (Xu et al., 2002), which dephosphorylate intracellular protein kinases such as LCK and ZAP70. These protein kinases are rapidly activated upon TCR interactions with a self-antigen, as continuously displayed by the major histocompatibility complex molecules on antigen-presenting cells, to initiate positive signalling cascades for immune-suppressive cytokine secretion and maintenance of normal immune homeostasis by Tregs (Fernández-Aguilar et al., 2023; Vahl et al., 2014; Kochi et al., 2010; Mkaddem et al., 2017). Thus, the FCRL3 cytoplasmic ITIM domain regulates downstream TCR signal transduction involved in the expansion and activation of Tregs. However, polymorphisms in the *FCRL3* gene can induce Tregs dysfunction, leading to rapid autoreactive T-cell proliferation that causes autoimmune diseases.

In this study, we investigated the associations between the *FCRL3* SNP rs7528684 (−169C/T) and RA in the Indian ethnicity. The study confirms that rs7528684 (−169C) is closely associated with RA in the Indian ethnicity. The statistical analyses (OR and RR) also suggest that the C/C genotype in the rs7528684 locus increases the risk of RA compared to the T/T and T/C genotypes. Similarly, the C allele frequency of rs7528684 was higher in RA patients than in the controls (58.6% vs. 43.1%). Similar results have been reported in other populations, such as Dutch, Japanese, and Taiwanese ethnic groups. Studies in the Dutch and Japanese ethnic groups revealed that the C/C genotype of rs7528684 is associated with RA and other autoimmune diseases (Thabet et al., 2007; Report, 2006; Kalantar et al., 2020). In the Taiwanese population, the C/C genotype frequency was high in destructive RA (Chen et al., 2010), whereas in the Iranian population, the C allele was associated with Hashimoto’s thyroiditis (Kalantar et al., 2020) and Behcet’s disease (Shahram et al., 2019). However, in the Spanish population, the C allele was found to be a protective allele against MS (Matesanz et al., 2008). We further investigated the impacts of rs7528684 (−169C) on *FCRL3* gene expressions in both controls and RA patients. For this, we isolated the mRNAs from the PBMCs and reverse-transcribed them to cDNA before quantifying the expression levels using qPCR. The results showed significantly higher *FCRL3* mRNA expression levels in RA patients than controls. Similarly, a comparison between the C/C and T/T genotypes revealed significantly higher mRNA expressions in RA patients with the C/C genotype than wild-type (T/T) genotype in the controls. The promoter region of a gene plays an essential role in prompting the binding of transcription factors that affect the target gene expression. The SNP rs7528684 (−169T>C) in the *FCRL3* promoter region (−169) away from the transcription initiation site, particularly the C/C genotype, enhances the binding affinities of the NF-κb family of transcription factors, leading to increased *FCRL3* expressions on the surfaces of the Tregs (Kochi et al., 2005; Agarwal et al., 2020; Swainson et al., 2010). Thus, elevated *FCRL3* expression disturbs the immune-suppressive roles of Tregs via the cytoplasmic motif (ITIM) and causes autoimmune diseases, including RA.

To investigate whether higher *FCRL3* mRNA levels influence *FOXP3* gene expression and FOXP3-dependent Treg immune-suppressive cytokine levels, we measured the *FOXP3* and *IL35* mRNA levels in early-onset RA patients and controls. The results showed significantly lower *FOXP3* mRNA expressions in RA patients than controls. Similarly, comparisons among the SNP rs7528684 genotypes showed significantly lower *FOXP3* mRNA levels in RA patients carrying the C/C and T/T genotypes. Upon TCR interactions with a self-antigen, the SRC family of kinases, including LCK and ZAP70, are involved in the initial activation of the TCR signal; this activation, in turn, triggers a downstream signalling cascade that ultimately activates phospholipase C (PLC γ) (Fernández-Aguilar et al., 2023; Yan et al., 2013). The PLC γ hydrolyses phosphatidylinositol-3,4-bisphosphate to produce the secondary messengers inositol-1,4,5-triphosphate (IP3) and diacylglycerol. IP3 binds to the IP3 receptor on the endoplasmic reticulum to release intracellular calcium (Ca^2+^) into the cytosol of Tregs. This Ca^2+^ helps in binding calmodulin to calcineurin phosphatase (Sana et al., 2021). The activated calcineurin phosphatase dephosphorylates the nuclear factor of activated T-cells (NFAT) to facilitate translocation into the nucleus, where it directly binds to the promoter region of *FOXP3* and initiates transcription in Tregs (Maruyama et al., 2012; Schmidt et al., 2012). Thus, Treg dysfunction mediated by rs7528684 (−169T>C) may lead to reduced *FOXP3* expression. However, the stability and expression of *FOXP3* are influenced by not only SNPs but also factors such as IL-6 and TNF-α and their associated signalling pathways (Roghani et al., 2023; Patel et al., 2021). This inflammatory environment in RA may explain the lower *FOXP3* expressions in RA patients even with the wild-type (T/T) genotype. Therefore, the precise mechanism underlying *FCRL3*-mediated *FOXP3* downregulation warrants further investigation. Furthermore, we performed Spearman’s rank correlation coefficient analysis between the *FCRL3* and *FOXP3* mRNA expressions, which revealed a significant negative correlation (r = −0.556, *p* = 0.0094, R^2^ = 0.3052). These findings suggest a pathogenic mechanism wherein increased *FCRL3* activity leads to downregulation of the key regulatory gene *FOXP3*, potentially impairing Treg functions in RA. Given the importance of *FOXP3*, a transcription factor that regulates the synthesis of anti-inflammatory cytokines in Tregs (Goldmann et al., 2024), we further measured the expression levels of *EBI3* and *P35* (subunits of IL-35) to evaluate the functional impacts of the observed decline in *FOXP3* mRNA expression as both subunits are directly transcribed by FOXP3. The results reveal a significant decrease in *IL-35* (*EBI3* and *P35*) mRNA levels in RA patients with the C/C genotype. In contrast, the higher *P35* levels observed in controls with the C/C genotype are likely attributable to the independent regulation of the P35 subunit. The P35 subunit is shared with other cytokines, such as IL-12, and its expression is regulated by different inflammatory signals (e.g., NF-kB and interferon regulatory factor pathways) (Wang et al., 2014). This may mask the genotype-specific effects of FCRL3 on P35 transcription. Meanwhile, EBI3 as the more specific subunit of IL-35 is more sensitive to *FCRL3*-mediated regulatory effects, as evidenced by its reduced expression in the C/C genotype. Recent studies have reported that abnormal expression of *IL-35* is directly associated with autoimmune diseases like SLE, MS, and type 1 diabetes. IL-35 helps restore the balance of the immune cells in RA and potentially reduces inflammation and joint damage.

The influences of the rs7528684 genotypes on serum IgG-RF and CRP levels were also evaluated in this study. IgG-RF and CRP are well-known biomarkers of inflammation that have been commonly detected in the serum of RA patients. IgG-RF is an autoantibody generated by the increased proliferation of autoreactive T and B cells to react against the Fc region of IgG. In the present study, we observed that IgG-RF-positive cases were significantly associated with the C/C genotype of rs7528684. This association may be attributed to the dysregulation of Tregs caused by the C/C genotype of rs7528684, which results in a higher number of autoreactive immune cells. Despite IgM-RF being the established diagnostic marker for RA, we screened the IgG-RF levels because IgM-RF can be positive in cases of infections (such as hepatitis or endocarditis), higher age, or presence of other autoimmune diseases. Most of the RA patients who participated in this study were over 40 years of age; therefore, measuring the IgG-RF levels effectively reduced the potential for obtaining false positive results. Furthermore, unlike IgM-RF, which is more associated with established RA and chronic disease progression, IgG-RF has been implicated in early inflammatory processes, including the amplification of immune responses through Fc receptor interactions. This aligns with our study focus on early-onset RA prior to the initiation of any NSAID/DMARD treatment (Tanaka, 2025; Ikari et al., 2006; Vignali et al., 2008; Nakamura et al., 1999). In autoimmune conditions, CRP is produced by the macrophages and other immune cells during the early stages of inflammation, which then leads to the production of proinflammatory cytokines (Pope and Choy, 2021). However, our results suggest that the *FCRL3* SNP rs7528684 is not significantly associated with CRP production.

This study demonstrates that the C/C genotype of the *FCRL3* SNP rs7528684 leads to upregulation of *FCRL3*, downregulation of *FOXP3* and *IL35*, and increased levels of IgG-RF, suggesting the mechanism underlying RA pathogenesis in the Indian population. However, the relationship between mRNA level and protein production in the immune system is not always direct because protein production is regulated by multiple factors beyond mRNA quantity. Therefore, additional assays such as flow cytometry, intracellular staining, and ELISA must be performed to study the molecular changes in *FCRL3, FOXP3*, and *IL-35* at the protein level, respectively. Further investigations are also required on cell-type-specific expressions to identify the cause of disease more precisely as these will reveal the distinct gene activities of various cell types.

## 5 Conclusion

Our study reveals an association between the *FCRL3* SNP rs7528684 and pathogenesis of RA in the Indian population. In particular, the C/C genotype of rs7528684 increases RA susceptibility by upregulating *FCRL3* gene expression and downregulating *FOXP3* and the anti-inflammatory cytokine *IL-35* (*EBI3* and *P35*)*.* Additionally, we observed a high prevalence of IgG-RF-positive cases in RA patients with the C/C genotype at the rs7528684 locus. Collectively, these findings suggest that the C/C genotype of the *FCRL3* SNP rs7528684 may contribute to RA by enhancing *FCRL3* expression. The increased *FCRL3* level is negatively correlated with *FOXP3* gene mRNA expression, which strongly indicates the pathogenesis of RA through the generation of autoreactive immune cells by dysregulation of immune-suppressive cytokine production in the Tregs.

## Data Availability

The original contributions presented in the study are included in the article/supplementary material; further inquiries can be directed to the corresponding author.
